# Perioperative peri-implant fracture after osteosynthesis for geriatric femoral pertrochanteric fracture with the linear compression integrated screw intramedullary nail system (INTERTAN™): a retrospective study

**DOI:** 10.1186/s13018-023-04441-w

**Published:** 2023-12-06

**Authors:** Chih-Yang Lai, Chang-Heng Liu, Po-Ju Lai, Yung-Heng Hsu, Ying-Chao Chou, Yi-Hsun Yu

**Affiliations:** Department of Orthopedic Surgery, Musculoskeletal Research Center, Chang Gung Memorial Hospital, Linkou Branch, and Chang Gung University, Taoyuan, Taiwan

**Keywords:** Bone screws, Femoral fractures, Femur, Periprosthetic fractures

## Abstract

**Background:**

Osteosynthesis for geriatric femoral pertrochanteric fractures using the linear compression integrated screw intramedullary nail system (INTERTAN™) has become popular. Nonetheless, cases of perioperative peri-implant fractures have been reported following this surgical technique. The factors responsible for this complication remain unclear. Therefore, we investigated perioperative peri-implant fracture risk factors and incidence, as well as overall outcomes, using the INTERTAN™ system for geriatric femoral pertrochanteric fractures.

**Methods:**

We retrospectively reviewed 98 consecutive patients with geriatric femoral pertrochanteric fractures after INTERTAN™ fixation, with at least a 12-month follow-up period between May 2020 and April 2022 at a single medical institute. The patients’ demographic characteristics, fracture pattern, quality of reduction, quality of fixation, nail length, morphology of the femur, and perioperative complications were recorded and analyzed.

**Results:**

Among the 98 patients, 92 achieved union during follow-up. Twelve perioperative peri-implant fractures (12.2%) were recorded, all of which occurred during or within 1 month of osteosynthesis. Except for one patient who underwent re-osteosynthesis, the others underwent nonoperative treatment, and all achieved union. Multiple regression analysis revealed morphology of the femur with low-lesser trochanter width (odds ratio (OR) 0.532, 95% confidence interval (CI) 0.33–0.86, *p* = 0.01) to be the only factor contributing to perioperative peri-implant fractures. When the Youden index was used, the optimal cut-off value was 20.2 mm of low-lesser trochanter width. Low-lesser trochanter width < 20.2 mm was found to be a potential factor causing perioperative peri-implant fractures (OR 17.81, 95% CI 1.67–19.76, *p* = 0.017).

**Conclusions:**

Morphology of the femur with a low-lesser trochanter width smaller than 20.2 mm was found to be the only potential contributor to perioperative peri-implant fractures when using INTERTAN™ for geriatric femoral pertrochanteric fractures. Care should be taken during osteosynthesis, focusing not only on the fracture site but also on the femoral cortex around the implant. Although perioperative peri-implant fractures were observed within one month following osteosynthesis, the majority of these cases were effectively treated without surgical intervention.

## Introduction

Geriatric femoral pertrochanteric fractures (GFPF) are among the most common operatively treated fractures in the geriatric population [1, 2]. The estimated global annual incidence of GFPF exceeds 1.6 million fractures, with a rising trend [3–5]. While the risk associated with anesthesia is a concern for older individuals, surgical intervention offers significant benefits over nonoperative treatment, particularly in terms of preventing complications arising from extended periods of immobility, facilitating the recovery of mobility, and reducing one-year mortality rates [6–10].

Traditionally, the preferred method for treating GFPFs has been the use of a sliding hip screw (SHS), owing to its high union rate, easy application, and cost-effectiveness [11–13]. However, there is a growing trend toward the use of intramedullary nails (IMN) for osteosynthesis [14–16]. Biomechanical studies and clinical evidence indicate that IMN surpass SHS in various aspects, including a shorter lever arm, better control over femoral shaft medialization, prevention of varus collapse, and reduced soft tissue dissection [17–20].

Over the years, advancements have been made in the design of IMN for treating GFPFs [21–25]. One such new IMN design is the linear compression integrated screw intramedullary nail system (INTERTAN™, Smith-Nephew Company), which incorporates a dual screw system that aims to provide anti-rotation of the femoral head (2-screw design) and achieve linear compression of the fracture gap (integrated screw design), thereby promoting bone union and avoiding complications such as proximal screw cut-off or cut-out from the femoral head [26, 27]. Studies have demonstrated that this new IMN design exhibits superior biomechanical outcomes compared with those of a single-lag screw nailing system [28–30].

However, as the utilization of IMN for GFPF treatment has increased, certain complications such as implant cut-out, implant breakage, and secondary femoral fractures have been documented [31–33]. Among them, perioperative peri-implant fractures (PPIF) are a notable concern. The reported incidence of PPIF ranges from 1 to 3%, varying depending on the specific implant design [34]. PPIF is a troublesome complication characterized by the occurrence of a new fracture shortly after the initial fracture, often necessitating repeated osteosynthesis. Despite its significance, few studies have evaluated the potential risk factors associated with PPIF following IMN treatment of GFPF.

This study aimed to accomplish the following objectives: (1) determine the incidence of PPIF, (2) analyze the potential risk factors associated with PPIF, and (3) report the outcomes following the occurrence of PPIF in osteosynthesis procedures for GFPF utilizing the linear compression integrated screw intramedullary nail system.

## Methods

We retrospectively reviewed the medical and radiological records from the institutional trauma registry of patients with GFPF who underwent osteosynthesis using the INTERTAN nail with the linear compression integrated screw intramedullary nail system (TRIGEN™ INTERTAN) between May 2020 and April 2022 at our institute. The review process was approved by our Institutional Review Board (IRB No. 202301241B0).

The INTERTAN nail was introduced in our institution in 2018. The available diameters of the INTERTAN nail were 10 mm, 11.5 mm, and 13 mm, with the 10 mm diameter being the smallest size. The inclusion criteria were patients aged > 60 years old who experienced femoral pertrochanteric fractures and underwent osteosynthesis using an INTERTAN nail, with complete medical and radiological follow-up for at least 12 months or until union. Fractures requiring revision osteosynthesis, those involving more than 2 parts of the femur, and those of pathological origin were excluded. Radiological follow-ups were conducted for all patients immediately after the surgery and at 1-, 3-, 6-, and 12-month intervals. The patients’ demographic profiles, fracture patterns, quality of reduction (QoR), quality of fixation (QoF), nail length (NL), morphology of the femur (MoF), and complications were recorded and analyzed.

### Applied classification

The pertrochanteric fracture was classified based on the Arbeitsgemeinschaft für Osteosynthesefragen (AO) classification system (2018 revision), including A1: simple pertrochanteric fracture; A2: multifragmentary pertrochanteric fracture or incompetence of the lateral wall (thickness ≤ 20.5 mm); and A3: intertrochanteric or reverse oblique fracture [26, 35]. Patients presenting with AO 31-A1 and -A2 fractures were primarily treated with short nails, specifically 180 mm and 200 mm in length. Conversely, individuals with fractures located in the intertrochanteric region accompanied by subtrochanteric extension, falling under the classification of AO 31-A3, were typically recommended for treatment using long nails as the standard implant option.

### Radiological evaluations

The QoR was assessed by comparing the neck-shaft angle of the operated site to that of the contralateral healthy hip on pelvic anteroposterior radiography and was classified as: “good” with less than 5 degrees difference from the normal side, “acceptable” with between 5 and 10 degrees of variation, and “poor” with more than 10 degrees of variation [36]. Other radiological parameters included varus (neck-shaft angle < 125°) or valgus reduction (neck-shaft angle > 125°) [37]. Positive, neutral, and negative medial femoral cortical supports were also evaluated [38, 39].

QoF was assessed based on the position of the lag screw using the tip-apex distance (TAD) and the Cleveland index [40, 41]. A critical TAD of 25 mm was established, as a TAD value of < 25 mm was considered protective against screw cut-out of the femoral head or failure [42]. The position of the lag screw tip was assessed using the Cleveland index [40]. As these evaluations were based on the single lag screw design of the implants, evaluations using the INTERTAN™ implant were conducted based on the upper screw in terms of the lag screw.

**Fig. 1:**
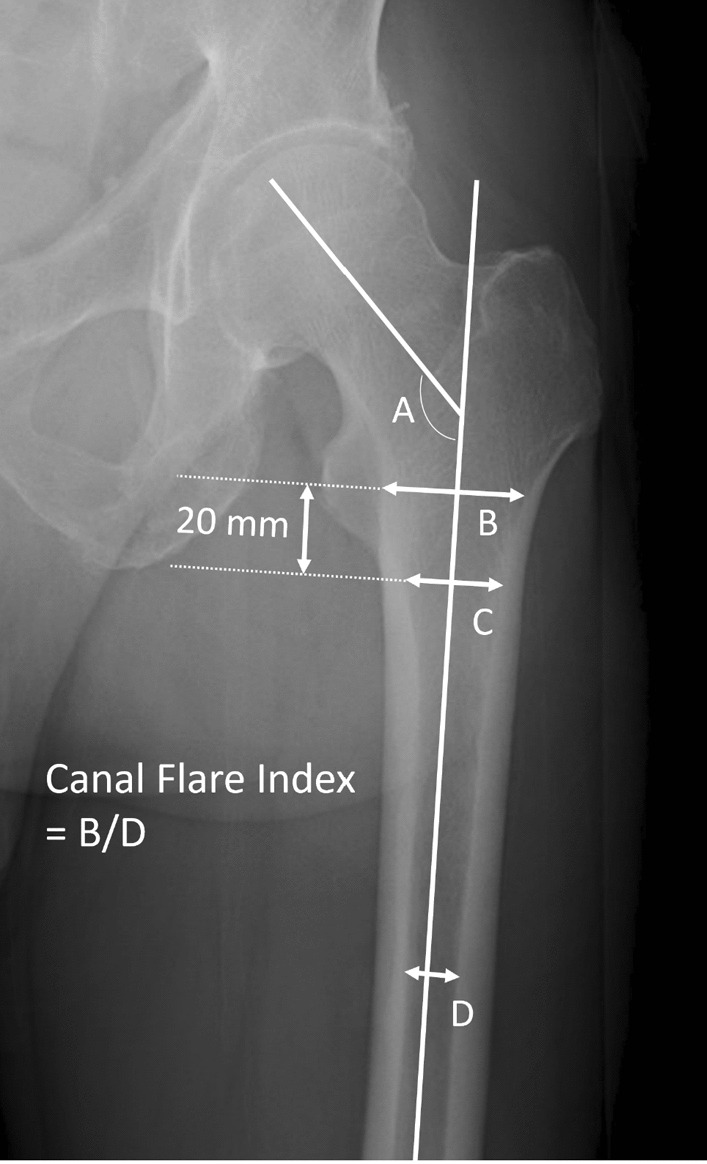
Radiologic measurements of the morphology of the femur. **A** Neck-shaft angle. **B** Cavity width at the mid-lesser trochanter level. **C** Cavity width 20 mm below the mid-lesser trochanter level (low-lesser trochanter width). **D** Diameter of the isthmus. The canal flare index was **B**/**D**

Because the geometry of the proximal femur may influence the PPIF, the MoF was also measured according to the radiographs, including the diameter of the isthmus (DI), lesser trochanter width (LTW), low-lesser trochanter width (LLTW) (cavity width 20 mm below the mid-lesser trochanter line), and canal flare index (the ratio of the DI in the anteroposterior view to the LTW) (Fig. 1) [43–46].

All the selected parameters were calibrated by using the corresponding nail size on the X-ray on the PACS system (Centricity Enterprise Web V3.0; GE Healthcare, Chicago, USA).

#### Rehabilitation protocol

Immediate walker-assisted weight-bearing ambulation is usually advised for GFPF. However, the rehabilitation protocol shifted more conservatively when a PPIF was identified. First, we considered whether the new fracture was stable or unstable with a nail present. If the fracture was unstable, re-do osteosynthesis with a long nail was necessary. In contrast, conservative treatment was chosen when the PPIF was stable. For patients with stable PPIF, a non-weight-bearing rehabilitation program was suggested for at least 6 weeks after the operation. When callus was detected on the follow-up X-ray, walker-assisted partial weight-bearing ambulation was begun. Finally, full weight-bearing ambulation was allowed when more callus formed, usually 3 months after the index surgery.

### Statistical analysis

Descriptive statistics were used to summarize cohort characteristics, with means and standard deviations reported for continuous variables, and frequencies and percentages for categorical variables. Continuous variables were compared using the Student’s t-test. Categorical variables were compared using Chi-square and Fisher’s exact tests. All analyses were performed using SPSS software (version 23.0; IBM Corp., Armonk, NY, USA). Statistical significance was determined using a two-tailed *p*-value less than 0.05.

## Results

**Fig. 2:**
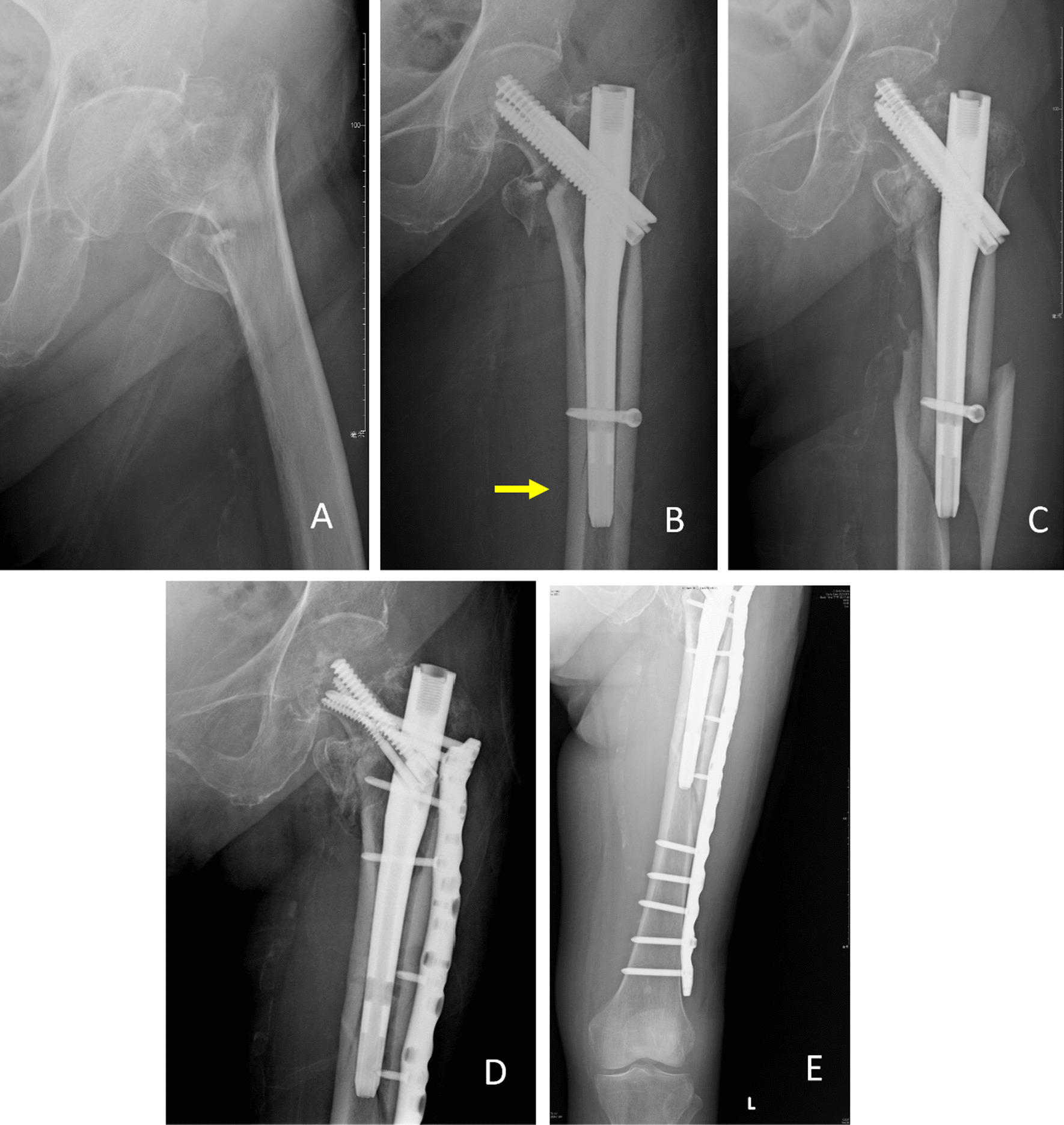
An illustration of perioperative peri-implant fracture (PPIF) that underwent revision osteosynthesis. A A femoral intertrochanteric fracture underwent osteosynthesis. **B** Nonoperative treatment for PPIF (arrow). **C** The fracture extended to the diaphysis 2 months later. **D** and **E** Revised osteosynthesis was performed

**Fig. 3:**
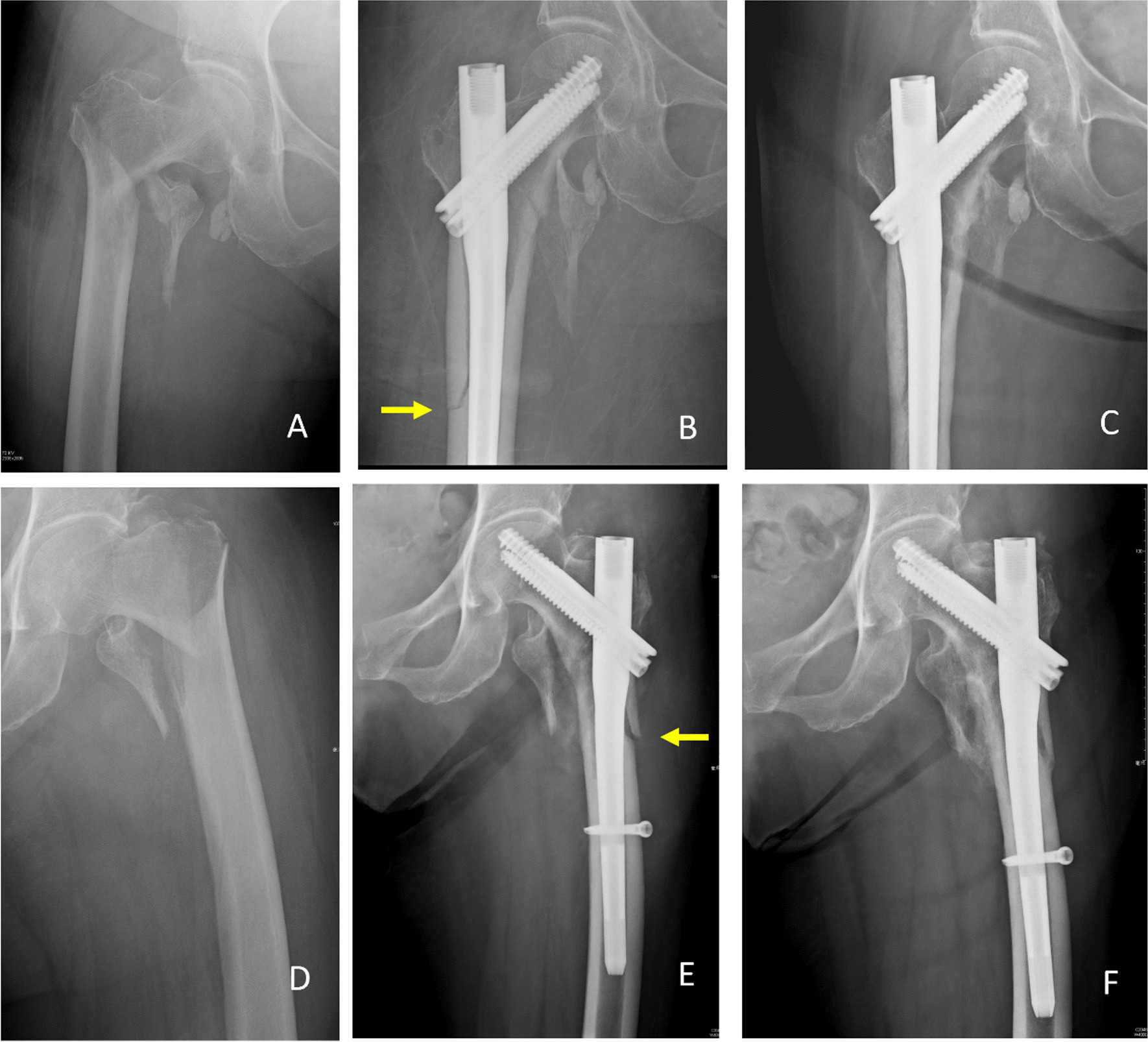
Illustrations of perioperative peri-implant fracture (PPIF) and related nonoperative treatment. **A** and **D** Femoral intertrochanteric fractures underwent osteosynthesis. **B** and **E** PPIF was noted over the lateral femoral cortex (arrow). **C ** and **F** Cortical continuity achieved 6 months later by nonoperative treatment

Ninety-eight patients who met the inclusion criteria during the study period were enrolled; their demographic data are shown in Table 1. While six patients showed nonunion of the fracture and required re-osteosynthesis, the union rate reached 93.9% (92 out of 98). Twelve patients experienced PPIF (incidence: 12.2%). Among the 12 cases of PPIF, 10 were identified during intraoperative fluoroscopic examination. The other 2 cases of PPIF were observed without new trauma by X-ray follow-up within 1 month postoperatively. One patient with an PPIF required revised osteosynthesis owing to the long extension of the fracture distal to the implant (Fig. 2). For the remaining 11 patients, union of the PPIF was achieved by nonoperative treatment within 9 months (Fig. 3).

**Table 1:** Demographic characteristics of 98 patients

Age (mean + SD) years	74.8 (SD 14.9)
*Sex*	
Male	40 (40.8%)
Female	58 (59.2%)
BMI (mean + SD)	23.7 (SD 4.6)
OTA classification^a^	
A1	10 (10.2%)
A2	73 (74.5%)
A3	15 (15.3%)
*Quality of reduction*	
*According to neck–shaft angle*	
Good	61 (62.2%)
Acceptable	25 (25.5%)
Poor	12 (12.2%)
*According to medial cortical support*	
Negative	9 (9.2%)
Neutral	45 (45.9%)
Positive	44 (44.9%)
*Quality of fixation*	
TAD (mm)	20.9 (SD 5.8)
*Position of lag screw (Cleveland index)*	
1/2/3	7 (7.1%)/35 (35.7%)/2 (2.0%)
4/5/6	2 (2.0%)/48 (49.0%)/2 (2.0%)
7/8/9	0/2 (2.0%)/0
Short nail	30 (30.6%)
Long nail	68 (69.4%)
*Morphology of the femur*	
Lesser trochanter width (mm)	30.4 (SD 4.1)
Low-lesser trochanter width (mm)	22.6 (SD 3.3)
Diameter of the isthmus (mm)	14.1 (SD 2.5)
Canal flare index	2.3 (SD 0.9)
*Complication*	
PPIF	12 (12.2%)
Nonunion	6 (6.1%)

*SD* Standard deviation, *BMI* Body mass index, *TAD* Tip-apex distance, *PPIF* Perioperative peri-implant fracture

^a^Based on the Arbeitsgemeinschaf für Osteosynthesefragen (AO/OTA) classification (2018 revision)

Table 2 compares selected factors between patients with (PPIF group) and without (NPPIF group) PPIF. The PPIF group had a higher BMI than the NPPIF group (23.2 versus 27.3, *p* = 0.005). The other significant finding was the MoF: the PPIF group had a smaller LTW (30.8 mm versus 28.0 mm, *p* = 0.026), smaller LLTW (23.2 mm versus 18.7 mm, *p* = 0.001), and smaller DI (14.4 versus 12.1, *p* = 0.002).

**Table 2:** Comparison of patients with and without PPIF following osteosynthesis for femoral pertrochanteric fracture with INTERTAN™

	PPIF (12)	NPPIF (86)	*p* value
Age (mean + SD)	77.7 (SD 9.7)	74.4 (SD 15.6)	0.488
Sex			0.069
Male	2 (16.7%)	38 (44.2%)	
Female	10 (83.3%)	48 (55.8%)	
BMI (mean + SD)	27.3 (SD 6.1)	23.2 (SD 4.2)	0.005
OTA classification^a^			0.646
A1	0	10 (11.6%)	
A2	12 (100%)	61 (71.0%)	
A3	0	15 (17.4%)	
*Quality of reduction*			
According to neck-shaft angle			0.362
Good	9 (75.0%)	52 (60.5%)	
Acceptable	3 (25.0%)	22 (25.6%)	
Poor	0	12 (14.0%)	
According to medial cortical support			0.103
	2 (16.7%)	7 (8.1%)	
Negative	8 (66.6%)	37 (43.0%)	
Neutral	2 (16.7%)	42 (48.8%)	
Positive			
*Quality of fixation*			
TAD (mm)	17.2 (SD 5.8)	21.5 (SD 5.6)	0.126
Cleveland index			0.182
1/2/3	1/2/2	6 /33/0	
4/5/6	0/7/0	2 /41/2	
7/8/9	0/0/0	0/2/0	
Nail length			0.334
Short nail	2 (16.7%)	28 (32.6%)	
Long nail	10 (83.3%)	58 (67.4%)	
*MoF*			
Lesser trochanter width (mm)	28.0 (SD 2.7)	30.8 (SD4.1)	0.026
Low-lesser trochanter width (mm)	18.7 (SD 2.6)	23.2 (SD3.0)	0.001
Diameter of isthmus (mm)	12.1 (SD1.2)	14.4 (SD2.5)	0.002
Canal flare index	2.4 (SD0.4)	2.3 (SD1.0)	0.693

*BMI* Body mass index, *TAD* Tip-apex distance, *MoF* Morphology of femur, *NPPIF* Non-perioperative peri-implant fracture, *PPIF* Perioperative peri-implant fracture

^a^Based on the Arbeitsgemeinschaf für Osteosynthesefragen (AO/OTA) classification (2018 revision)

**Fig. 4:**
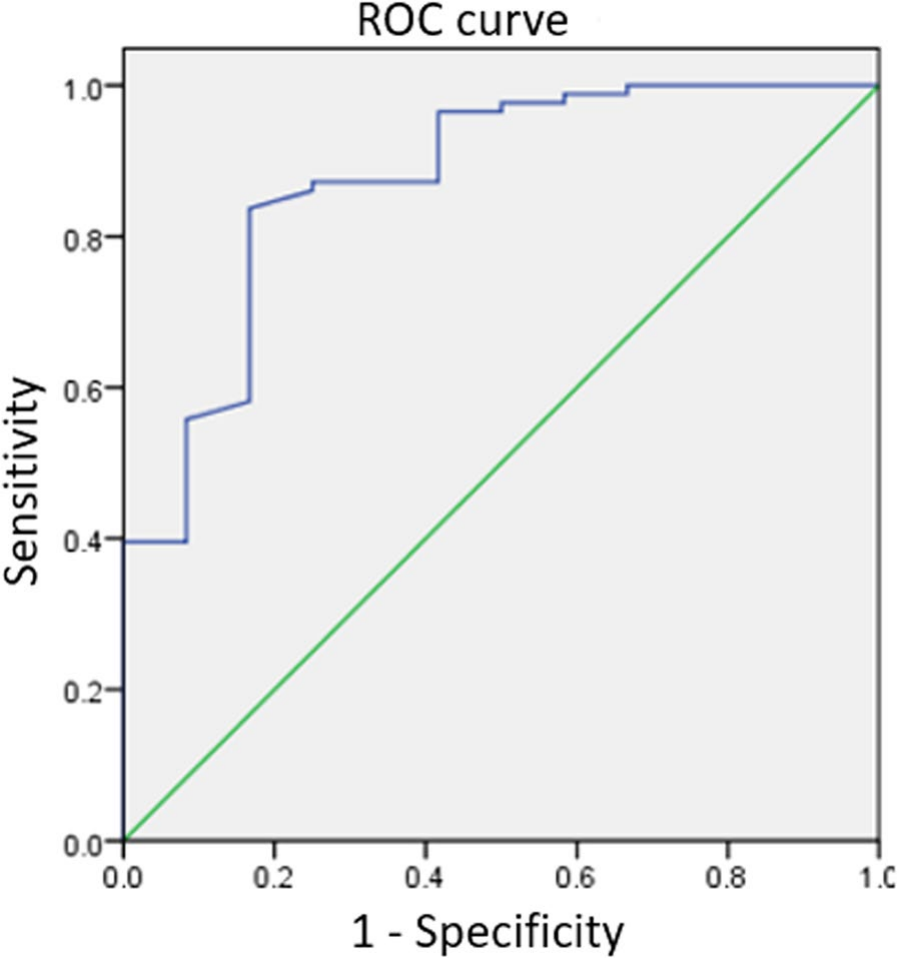
ROC curve for low-lesser trochanter width attempts to predict the complication of peri-implant fracture. *ROC* receiver operating characteristic area under the curve: 0.874

Because a relatively small number of patients was enrolled, a stepwise method of multivariate logistic regression analysis was applied (Table 3), which resulted in only one significant independent predictor: LLTW (odds ratio (OR): 0.532, 95% confidence interval (CI) = 0.33–0.86, *p* = 0.01). The area under the curve was 0.874 (OR 17.81, 95% CI 1.67–19.76, *p* = 0.017) on the receiver operating characteristic curve. The Youden index revealed that the optimal cutoff value of LLTW was 20.2 mm (Fig. 4).

**Table 3:** Results of multiple logistic regression analysis for PPIF

Risk factors			
	OR	Estimated 95% CI	*p* value
BMI	1.093	0.93–1.27	0.264
Lesser trochanter width	1.152	0.79–1.68	0.464
Low-lesser trochanter width	0.532	0.33–0.86	0.010
Diameter of isthmus	0.827	0.42–1.63	0.583
Canal flare index	0.736	0.45–11.98	0.736
Low-lesser trochanter width < 20.2 mm	17.81	1.67–19.76	0.017

*OR* Odds ratio, *CI* Confidence interval, *BMI* Body mass index, *PPIF* Perioperative peri-implant fracture

## Discussion

Only a few studies have addressed the occurrence of PPIF and its associated factors in Asian populations using a single implant for GFPF [47]. With reference to this gap in the literature, our study revealed that the incidence of PPIF in patients with GFPF after INTERTAN™ fixation was 12.2%, which was relatively higher than the rates reported by other studies (1 to 3%) [34, 47, 48]. Moreover, through femoral morphology analysis, we identified a specific potential factor, namely, the width of the LLTW, which might be responsible for causing PPIF. We found that a critical LLTW width of less than 20.2 mm could significantly increase the risk of PPIF.

Although employing the SHS system to treat GFPF has a long history, using a proximal femoral intramedullary nail for such fractures has been shown to offer several advantages [14, 18]. However, accompanying complications such as PPIF after nailing may increase. A systematic review reported that the incidence of secondary fractures around the nail is 1.7% [34]. Additionally, Muller et al. reported that peri-implant fractures occur within the proximal femoral nails much more frequently than in dynamic hip screws [33], and the position of the lag screw is a potential factor associated with PPIF. Helwig et al. conducted a laboratory study using finite element analysis and discovered that cranial positioning of the lag screw increased stress on the proximal femur, contributing to a higher risk of peri-implant fracture [49]. In this study, the lag screws were positioned at the center-center or inferior-center in the femoral head, adhering to the criteria of TAD (less than 25 mm) and the Cleveland index, which aimed to prevent migration of the lag screw and facilitate fracture union. Consequently, the present study did not find evidence supporting the influence of an inappropriate lag screw position on PPIF occurrence.

The potential effect of short or long intramedullary nails on PPIF remains uncertain. Frisch et al. conducted a comparative study involving 169 patients and reported a higher peri-implant fracture rate in short nails than in long nails [50]. However, other studies have indicated no difference in the incidence of PPIF between short and long nails [34, 47, 51]. In our study, there was no significant difference in the PPIF rates between short and long nails. Of the 12 PPIFs, 11 occurred near the subtrochanteric area and were effectively treated using nonoperative methods. However, in one case, re-osteosynthesis was necessary because the short nail lost its stability. This observation leads us to advocate for the use of long nails in high-risk patients (those with a narrow LLTW measuring less than 20.2 mm). This can improve stability and reduce the need for re-do surgery if PPIF occurs.

Irrespective of the design and geometry of the intramedullary nail, patient factors such as increased BMI might have influenced the PPIF. However, the cause of the relatively high incidence of PPIF after intramedullary nail fixation remains unclear. Based on our analysis, a potential factor contributing to PPIF could be the discrepancy between the bone mineral density and the INTERTAN™ design. Notably, Asian populations have been observed to possess a greater cortical thickness in the proximal femur compared to that in Caucasian individuals [52]. Thiesen et al. reported that the proximal isthmus distance was relatively consistent but was more proximal in Asians than in Caucasians [53]. The INTERTAN™ nail is specifically designed with a proximal trapezoidal shape to enhance strength and stability during flexion and extension of the femur. However, due to the proximity of the isthmus and thickness of the femoral cortex in the Asian population, the insertion of a trapezoidal proximal nail may add additional stress to the proximal femoral cortex, theoretically leading to an increased risk of PPIF in this population.

Despite efforts to minimize bias, our study has certain limitations. First, its retrospective design introduced the risk of recall bias, and the relatively small sample size from a single institution may have led to potential bias. The limited sample size also constrained our ability to identify the independent risk factors for PPIF. Additionally, the inclusion of several orthopedic surgeons in the study might have introduced bias owing to variations in surgical techniques. Furthermore, quantification of femoral geometry relies on radiography instead of more precise methods, such as computed tomography. The position of the patient during the X-ray examinations may have also influenced the results. Moreover, certain anatomical details, such as femoral bowing, were not obtained, and these anatomical variations may play a role in PPIF development. Finally, the fracture pattern of PPIF was evaluated only by X-rays. A computed tomography scan would be a better examination tool to clarify the pattern and orientation of the fracture. This would allow for better assessment of treatment options (conservative treatment or re-do osteosynthesis). Further prospective studies with larger sample sizes and well-designed image interpretations, such as computed tomography, should be conducted. This would help gain a deeper understanding of the condition and its potential risk factors.

## Conclusions

While INTERTAN™ has shown a high union rate in treating GFPF, it is crucial to be vigilant regarding the occurrence of PPIF. A narrow LLTW measuring less than 20.2 mm emerges as a potential risk factor for an unforeseen fracture. Care should be taken during osteosynthesis, especially during insertion of the nail, not only concentrating on the fracture site but also considering the entire femur surrounding or distal to the implant. Despite the occurrence of PPIF, most cases can be effectively managed without surgical intervention. Nonoperative approaches have shown promise in managing PPIF with favorable outcomes.

## Data Availability

The datasets used and analyzed during the current study are available from the corresponding author on reasonable request.

## Abbreviations

AO: Arbeitsgemeinschaft für Osteosynthesefragen
CI: Confidence interval
DI: Diameter of the isthmus
GFPF: Geriatric femoral pertrochanteric fractures
IMN: Intramedullary nails
INTERTAN: Integrated screw intramedullary nail system
LLTW: Low-lesser trochanter width
LTW: Lesser trochanter width
MoF: Morphology of the femur
NL: Nail length
NPPIF: Without PPIF
OR: Odds ratio
PPIF: Perioperative peri-implant fractures
QOF: Quality of fixation
QoR: Quality of reduction
SHS: Sliding hip screw
TAD: Tip–apex distance
